# An Unusual Case of GRIN2A Mutation Presenting as Progressive Limbic Encephalopathy in an Adult

**DOI:** 10.7759/cureus.63046

**Published:** 2024-06-24

**Authors:** Dorsa Heydarlou, Arya Asghari, Shawyon Ezzati, Mariam Khalil, Shahnawaz Karim, Forshing Lui

**Affiliations:** 1 Neurology, California Northstate University College of Medicine, Elk Grove, USA; 2 Neurology, Kaiser Permanente, Sacramento, USA; 3 Clinical Sciences, California Northstate University College of Medicine, Elk Grove, USA

**Keywords:** neuropsychiatric disorders, progressive cognitive decline, nmda receptor, limbic encephalopathy, grin2a mutation

## Abstract

The glutamate ionotropic receptor NMDA (N-methyl-D-aspartate) type subunit 2A gene (*GRIN2A*) encodes the GluN2A subunit of NMDA receptors, which are essential for synaptic plasticity and memory consolidation. Mutations in *GRIN2A *can disrupt these processes, often affecting the pediatric population and causing various neurological disorders characterized by epilepsy, intellectual disability, and aphasia, among other neuropsychiatric findings. We report an unusual presentation of adult-onset *GRIN2A* mutation-associated progressive limbic encephalopathy (LE), characterized by rapidly progressive cortical atrophy, seizures, aphasia, and neuropsychiatric abnormalities, which ultimately led to the patient’s sudden demise. Further research into *GRIN2A* mutations will improve our understanding of such presentations, guiding enhancements in diagnostic methods and therapeutic approaches.

## Introduction

The *GRIN2A* gene, which stands for glutamate ionotropic receptor NMDA (N-methyl-D-aspartate) type subunit 2A, encodes the GluN2A subunit of NMDA receptors. These receptors play a crucial role in initiating signaling cascades involving calcium ion influx, an essential process for synaptic plasticity and memory consolidation [1,2]. Mutations in *GRIN2A* can disrupt these processes and often affect children, leading to a variety of neurological disorders [3,4]. Clinical presentations can include epilepsy, intellectual disability, aphasia, schizophrenia, muscular hypotonia, movement disorders, and autism spectrum disorder [3,5,6]. Brain MRI of individuals with *GRIN2A* mutations generally reveals mild abnormalities, including focal cortical dysplasia, dysplastic corpus callosum, impaired myelination, hippocampal hyperintensity, hippocampal sclerosis, and generalized atrophy [4,7,8].

Limbic encephalopathy (LE) encompasses a broad range of disorders affecting the limbic system, which is responsible for memory, emotion, and behavior. Its etiologies include autoimmune responses [9-11], infection [12], paraneoplastic syndromes [13], metabolic disorders [14], and neurodegenerative diseases [15,16]. Its clinical manifestations can be acute, subacute, or chronic, and include short-term memory deficits, epilepsy, aphasia, abnormal behaviors, delirium, and other psychiatric abnormalities [9,11,13]. Clinical assessment involves MRI to detect hyperintensities or atrophy in limbic structures, cerebrospinal fluid (CSF) analysis, and antibody screening, among other diagnostic modalities, based on the patient’s history and presentation [10,17]. Diagnosis is often delayed as symptoms can overlap with other neurodegenerative and epileptic disorders. Treatment is generally supportive and aimed at managing symptoms, such as seizures and psychiatric disturbances, in addition to addressing underlying causes such as autoimmune processes and cancer using immunotherapy [14-16].

Although *GRIN2A* mutations can present with epilepsy, aphasia, and various neuropsychiatric manifestations, they have not previously been associated with LE. This case report describes a rare presentation of *GRIN2A* mutation-associated LE in an adult.

## Case presentation

A 47-year-old Mexican American male with a history of bilateral sensorineural hearing loss managed with hearing aids, developed progressive cognitive decline, speech abnormalities, hallucinations, and fluctuating behavioral disturbances over several months. The patient had been healthy before a trip to Mexico in July 2018, during which he experienced an acute-onset fever of 39 ℃ and was diagnosed with an ear infection. He was evaluated by a physician in Mexico and prescribed a course of antibiotics. Shortly afterward, his family noticed decreased verbal communication, agitation, and excessive fatigue.

He was reevaluated in Mexico, where a CT of the head and echocardiogram revealed no abnormalities. He subsequently experienced a witnessed generalized tonic-clonic seizure, characterized by right-sided convulsions and right-sided head deviation. He was given diazepam, though he continued to exhibit minimal verbal output. Subsequently, he developed insomnia, visual hallucinations, paranoid delusions, and incoherent speech. Diazepam was discontinued, as it was suspected to be contributing to his behavioral abnormalities. Treatment with haloperidol was initiated, resulting in minor improvement of his visual hallucinations. however, his aphasia continued to worsen, characterized by difficulty in word retrieval and echolalia. He had significant difficulty understanding and following commands, prompting an evaluation by a neurologist due to concerns of a cerebrovascular accident or toxic encephalopathy. A lumbar puncture was performed, and CSF analysis yielded no evidence of infection or inflammation. Herpes simplex virus polymerase chain reaction, gram stain, and culture were normal, as were laboratory results for vitamin B12, ammonia, calcium, complete blood count (CBC), and alanine aminotransferase.

**Figure 1:**
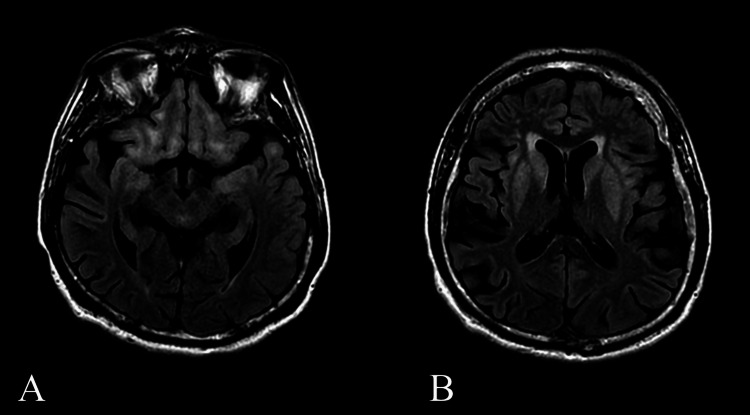
MRI findings in July 2018 A, B: axial T2W-FLAIR MRI taken in July 2018 depicting bilaterally symmetric hyperintensities in the (A) orbital frontal lobes, medial temporal lobes, (B) subinsular cortical areas, and basal ganglia with temporal cortical atrophy T2W: T2-weighted; FLAIR: fluid-attenuated inversion recovery; MRI: magnetic resonance imaging

After returning to the United States, the patient's neuropsychiatric status continued to decline with fluctuating severity. His symptoms returned to near baseline function for periods at a time, as he was able to assist with household tasks and return to work as a railcar assembly technician. He was examined by a neurologist shortly after returning from Mexico in July 2018 and received supportive care for encephalopathy of unclear etiology. T2-weighted (T2W) and fluid-attenuated inversion recovery (FLAIR) MRI studies revealed extensive bilateral and symmetric hyperintensities involving the orbital frontal lobes, medial temporal lobes, subinsular cortical areas, and basal ganglia. Additionally, temporal cortical atrophy was noted, an unusual finding for his age. No contrast enhancement was observed (Figure 1). In October 2018, electroencephalography (EEG) findings revealed an epileptiform focus in the left temporal region. The patient was reevaluated and prescribed levetiracetam, the dosage of which was increased in November 2018. Over the next several months, his condition deteriorated despite transient periods of improvement.

In April 2019, the patient presented to the emergency department due to altered mental status and personality changes, attributed to the increased dosage of levetiracetam. He was hospitalized, started on phenytoin, and weaned off levetiracetam, resulting in a brief improvement in symptoms. Over the next week, he experienced increasing confusion, social withdrawal, anxiety, and depression. He also exhibited behavioral changes towards family and hospital staff, such as inappropriate sexual advances, homicidal behavior, and neglecting personal hygiene. Further neurological evaluation suggested significant language impairment. He demonstrated the ability to follow simple commands and name basic objects; however, he demonstrated non-fluent aphasia with preservation. He was able to read and write simple sentences, though with notably slow speed and considerable hesitancy. His laboratory results comprising a lipid panel, CBC, comprehensive metabolic panel, thyroid-stimulating hormone, vitamin B12, vitamin B9, and HbA1c were within normal limits. At this time, differential diagnoses included acute toxic encephalopathy and acute encephalitis. Phenytoin was discontinued and supportive care, including speech therapy, was continued.

**Figure 2:**
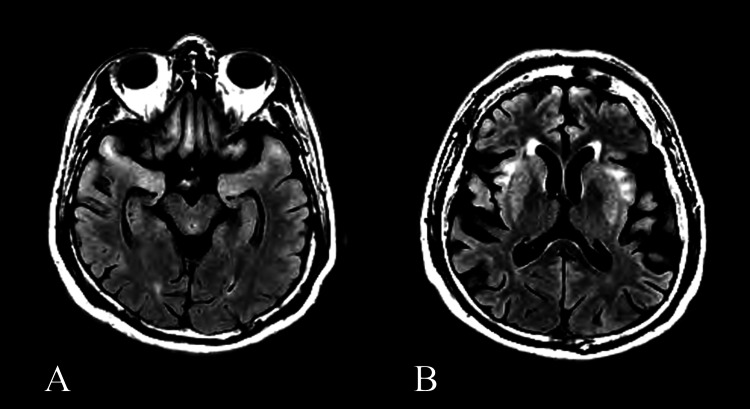
MRI findings in December 2019 A, B: axial T2W-FLAIR MRI taken in December 2019 depicting bilaterally symmetric hyperintensities in the (A) orbital frontal lobes, medial temporal lobes, (B) subinsular cortical areas, and basal ganglia. (B) A notable increase in cortical atrophy is observed in the frontal and temporal lobes T2W: T2-weighted: FLAIR: fluid-attenuated inversion recovery: MRI: magnetic resonance imaging

In December 2019, the patient’s condition deteriorated precipitously, confining him to his home with no social activity despite comprehensive supportive therapy, ultimately leading to his sudden demise. Repeat FLAIR MRI sequence showed worsening hyperintensity and cortical atrophy (Figure 2). Postmortem genetic analysis identified a *de novo* missense mutation in the *GRIN2A* gene.

## Discussion

This case report highlights an atypical presentation of *GRIN2A* mutation-associated progressive LE in an adult. *GRIN2A* mutations, more commonly observed in pediatric populations, often present with epilepsy, intellectual disability, aphasia, muscular hypotonia, and movement disorders [3]. The rapid neuropsychiatric decline and significant atrophy observed in this patient, despite intermittent improvements, shed light on the aggressive nature of LE in the context of *GRIN2A* mutations and suggest a severe phenotype of *GRIN2A* mutation-related encephalopathy.

While *GRIN2A* mutations typically manifest in children, our case demonstrates that adults can experience similar symptoms, including behavioral abnormalities, aphasia, and seizures. Previous reports have described *GRIN2A* mutation-associated MRI changes as mild or nonexistent, which contrasts with our findings of extensive bilateral hyperintensities and temporal cortical atrophy, as depicted in Figures 1-2 [4,7,8]. This suggests a broader spectrum of MRI presentations in *GRIN2A* mutations, providing new insights into its pathological findings. Interestingly, despite significant basal ganglia involvement, the patient did not exhibit expected extrapyramidal symptoms, thereby suggesting a unique pathological process in *GRIN2A* mutations.

Progressive LE serves as the likely diagnosis, though differential diagnoses of toxic encephalopathy and leukoencephalopathy were explored. Toxic encephalopathy was considered due to bilateral and symmetric MRI findings; however, the patient's history did not indicate toxic injury. Leukoencephalopathy, which involves the white matter of the central nervous system, was deemed unlikely given the predominance of gray matter involvement [18]. Based on the selective involvement of limbic structures such as the hippocampus and amygdala, as well as an epileptiform focus in the left temporal region identified by EEG, progressive LE associated with *GRIN2A* mutation was the primary diagnosis, which was further supported by the patient’s neuropsychiatric symptoms and predominantly gray matter changes. The complex nature of this case is compounded by fragmented medical records from Mexico and the United States, limiting comprehensive longitudinal assessment. Also, it remains unclear how much of the patient’s symptoms were attributable to brain lesions as opposed to psychiatric manifestations, thus complicating diagnosis and management.

Ultimately, the presentation of progressive LE associated with *GRIN2A* mutation suggests that the umbrella of *GRIN2A* mutation-related disorders may be broader than previously understood. The patient’s rapid neuropsychiatric and functional decline emphasizes the significant impact *GRIN2A* mutations can have when presenting in adulthood. Previous literature has found that *GRIN2A* mutations can be inherited, a unique characteristic among pathological GRIN gene family mutations [3]. This finding, along with the observations from the presented case, demonstrates the importance of considering genetic testing in atypical neurodegenerative presentations.

## Conclusions

We discussed an unusual presentation of adult-onset *GRIN2A* mutation associated with progressive LE. The rapid progression characterized by significant cortical atrophy, seizures, aphasia, and neuropsychiatric abnormalities necessitates further studies to fully comprehend the wide spectrum of *GRIN2A* mutations. Insights from this case suggest that, in addition to the individual genetic background and environmental influences, the specific nature and location of the *GRIN2A* mutation may determine the resultant clinical phenotype. Continued research into *GRIN2A* mutations will enhance our understanding of associated neurological manifestations, thereby improving diagnostic methods and therapeutic approaches.
